# Regulating Nrf2-GPx4 axis by bicyclol can prevent ferroptosis in carbon tetrachloride-induced acute liver injury in mice

**DOI:** 10.1038/s41420-022-01173-4

**Published:** 2022-09-07

**Authors:** Tianming Zhao, Zihan Yu, Lei Zhou, Xiaoyu Wang, Yangyang Hui, Lihong Mao, Xiaofei Fan, Bangmao Wang, Xingliang Zhao, Chao Sun

**Affiliations:** 1 Department of Gastroenterology and Hepatology, Tianjin Medical University General Hospital, Anshan Road 154, Heping District, 300052 Tianjin, China; 2 Department of Gastroenterology, Nanjing Drum Tower Hospital, Chinese Academy of Medical Science & Peking Union Medical College, Zhongshan Road 321,Gulou District, 210008 Nanjing, Jiangsu China; 3 Tianjin Institute of Digestive Disease, Tianjin Medical University General Hospital, Anshan Road 154, Heping District, 300052 Tianjin, China; 4 Tianjin Neurological Institute, Tianjin Medical University General Hospital, Anshan Road 154, Heping District, 300052 Tianjin, China; 5 Department of Gastroenterology, Tianjin Medical University General Hospital Airport Hospital, East Street 6, Tianjin Airport Economic Area, 300308 Tianjin, China

**Keywords:** Cell death, Hepatotoxicity, Pharmacology

## Abstract

Hepatocellular death is a sensitive parameter for detecting acute liver injury (ALI) of toxic, viral, metabolic, and autoimmune origin. Ferroptosis has recently been implicated in carbon tetrachloride (CCl_4_)-induced ALI. However, the underpinning mechanism and mechanistic basis remain elusive. In this study, bicyclol, a proprietary hepatoprotectant in China, and ferroptosis-specific inhibitor ferrostatin-1 (Fer-1) were administered in CCl_4_-injured mice. A panel of ferroptosis-related markers, including mitochondria morphology, reactive oxygen species production, protein adducts in response to lipid peroxidation, and key modulators of ferroptotic process, was determined in vivo. Erastin-treated L-O2 hepatocytes were transfected with glutathione peroxidase 4 (GPx4) or nuclear factor erythroid 2-related factor 2 (Nrf2) siRNA to delineate the pathway of bicyclol against ferroptosis in vitro. As a result, CCl_4_ led to iron accumulation, excessive reactive oxygen species production, enhanced lipid peroxidation, and characteristic morphological changes in mitochondria, along with a decrease in GPx4 and xCT protein levels in ALI mice liver, all of which were generally observed in ferroptosis. The use of Fer-1 further corroborated that ferroptosis is responsible for liver damage. Bicyclol exerted its hepatoprotection by preventing the aforesaid ferroptotic process. Furthermore, bicyclol alleviated erastin-induced cellular inviability, destruction, and lipid peroxidation in vitro. Knockdown of GPx4 diminished these protective activities against perturbations associated with ferroptosis in L-O2 hepatocytes. Additionally, Nrf2 silencing drastically reduced GPx4 levels, and further impeded the medicinal effects of bicyclol. In summary, positively regulating Nrf2-GPx4 axis by bicyclol can prevent ferroptosis in CCl_4_-induced ALI in mice.

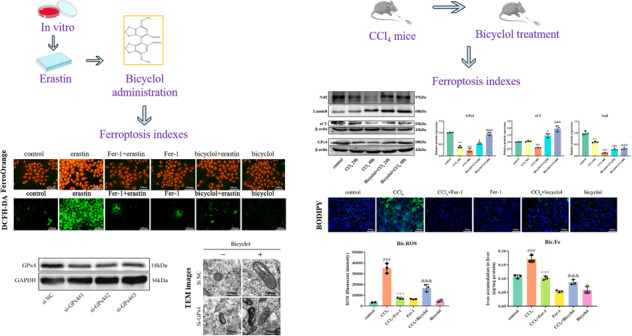

## Introduction

Hepatocellular death is widely present in almost all kinds of human liver diseases and regarded as a sensitive parameter to detect acute liver injury (ALI) of toxic, viral, metabolic as well as autoimmune origin [1]. Distinct modes of cell death such as necrosis, apoptosis, and necroptosis evoke a specific response and promote liver disease progression, which is manifested by the subsequent development of inflammation and ultimate fibrosis, cirrhosis, or liver failure in circumstance. Carbon tetrachloride (CCl_4_)-induced ALI is one of the best-characterized models of xenobiotic hepatotoxicity, which is broadly used for clarifying the protective/anti-hepatotoxic properties of the agent. The detrimental impacts of CCl_4_ can be interpreted as oxidative damage due to lipid peroxidation stemming from the conversion of CCl_4_ to free radicals [2]. These toxic radicals trigger multiple pathobiological processes, such as apoptosis, autophagy, and ferroptosis. Notably, our previous work clearly demonstrated that impaired autophagic flux is intimately involved in the pathogenesis of CCl_4_-induced ALI, whereas bicyclol represents protective potential with the induction of autophagy and concomitant antioxidative stress and anti-inflammatory response [3]. However, in-depth investigation is warranted to delineate the central role of ferroptosis in the model of ALI, which may pave the way for developing a promising therapeutic strategy.

Ferroptosis is deemed to be a novel type of regulated cell death (RCD). The process of ferroptosis is instigated by iron accumulation, reactive oxygen species (ROS) generation, and subsequent lipid peroxidation [4]. This modality may interconnect with other types of RCD via the release of damaging molecules, thus resulting in tissue injury and organ dysfunction [5]. Glutathione peroxidase 4 (GPx4) and xCT (subunit solute carrier family 7 member 11/SLC7A11) serve as key ferroptotic pathway molecules, which are responsible for the cellular import of cystine chronological reduced to cysteine and biogenesis of glutathione (GSH). The latter constitutes the main defense system against ROS accumulation. As a result, cell death attributed to ferroptosis can be triggered by either GSH depletion or GPx4 inactivation. Meanwhile, commonly used inhibitors of ferroptosis are suggested to eliminate lipid radicals such as ferrostatin-1 (Fer-1) and liproxstatin-1 [6].

Nuclear factor erythroid 2-related factor 2 (Nrf2) is a transcriptional factor that contributes to the regulation of intracellular redox homeostasis [7]. Intriguingly, two potential targets whose inactivation may elicit ferroptosis, namely xCT and GPx4, are well documented to be modulated by Nrf2. In the literature, the expression level of Nrf2 correlates with ferroptosis sensitivity, as cancer cells with downregulated Nrf2 are susceptible to pro-ferroptotic pharmaceuticals, whilst overexpression of Nrf2 prevents the initiation and execution of ferroptosis. Collectively, it is conceivable to foster Nrf2 activity as a viable therapeutic target for treating ferroptosis-related diseases, since emerging evidence has confirmed the pivotal role of Nrf2 in ferroptosis process [8, 9].

**Fig. 1:**
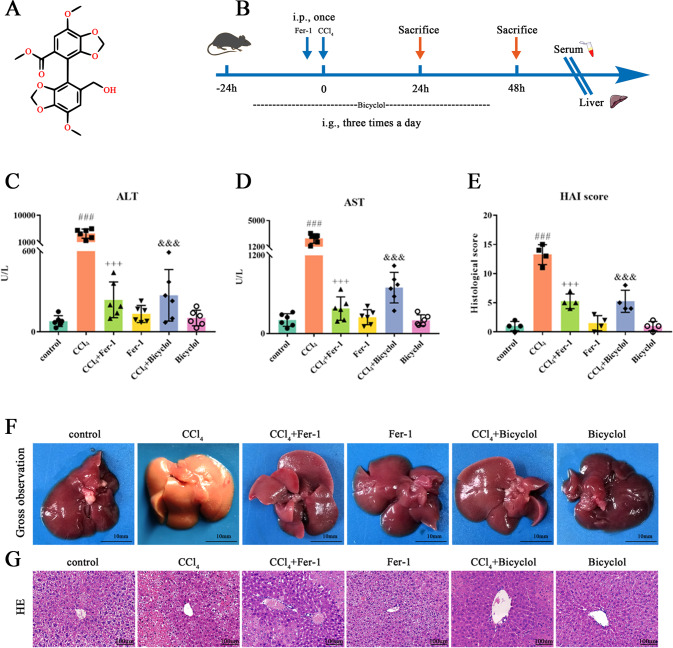
Bicyclol protects against CCl_4_-induced liver injury in vivo. **A** The chemical structure of bicyclol. **B** Schematic diagram of the experimental procedures. **C**, **D** Serum levels of ALT/AST were determined. **E** Histological changes were determined according to HAI score. **F** Macroscopic examinations were shown. **G** Microscopic changes were determined according to H&E staining. Data were expressed as mean ± SD. ^###^*p* < 0.001 vs control group, ^+++^*p* < 0.001 vs CCl_4_ group, ^&&&^*p* < 0.001 vs CCl_4_ group.

Bicyclol, an innovative proprietary with intellectual property in China, is derived from traditional Chinese medicine *Schisandra chinensis (Wuweizi)* (Fig. 1A) [10]. Bicyclol has versatile therapeutic effects, including antiviral replication, antioxidative stress, antifibrosis, anti-liver destruction, and stimulation of protein synthesis in hepatocytes [11]. As bicyclol can affect divergent types of RCD relevant to its hepatoprotection in the literature, thus it is our reasonable speculation and purpose to perform experiments to clarify the pharmacological activities of bicyclol against ferroptosis responsible for liver injury.

In this study, substantial data confirmed that hepatocellular ferroptosis contributes to the liver damage induced by CCl_4_ exposure. Further evidence demonstrated that bicyclol confers medicinal effects by preventing ferroptosis to ameliorate ALI via Nrf2-GPx4 axis.

## Results

### Ferroptosis is responsible for CCl_4_-induced ALI

First of all, we have to determine whether ferroptotic cascade is involved in the pathogenesis of CCl_4_-induced liver injury. In alignment with our previous findings, we selected liver injury at 48 h taking into consideration the most apparent histopathological changes. As shown in Fig. 1C, D, treatment with Fer-1, a lipophilic radical trap especially targeting ferroptosis, significantly reduced serum ALT/AST levels elevated by CCl_4_ in comparison to the control group. Consistently, the Knodell score for CCl_4_ group increased to 13.3 ± 0.9, which was considerably ameliorated by Fer-1 treatment (Fig. 1E). The macroscopic examination and H&E staining also demonstrated that Fer-1 alleviates liver damages and cell death of mice subjected to CCl_4_ (Fig. 1F, G). Similarly, the medicinal effects have been ascertained in the bicyclol treatment group, supporting the hepatoprotective action of bicyclol.

**Fig. 2:**
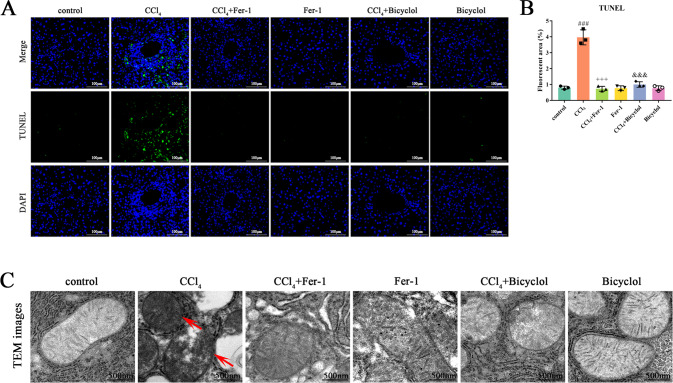
Bicyclol inhibits CCl_4_-induced hepatocellular ferroptosis in vivo. **A** The dead hepatocytes were monitored by TUNEL staining in fixed liver tissue sections. Representative images were shown. **B** Quantification of TUNEL-positive cells was shown. **C** TEM was used to detect the mitochondrial morphology of ferroptotic cells in liver tissue (red arrow). Data were expressed as mean ± SD. ^###^*p* < 0.001 vs control group, ^+++^*p* < 0.001 vs CCl_4_ group, ^&&&^*p* < 0.001 vs CCl_4_ group.

TUNEL-positive cells were significantly increased due to CCl_4_ administration, whereas both Fer-1 and bicyclol mitigated these cell death in the mice liver (Fig. 2A, B). To further clarify the central role of ferroptosis in CCl_4_-injured mice, we used TEM to observe the mitochondrial morphology in hepatic parenchyma. Of note, ferroptotic cells are characterized by structural alteration in mitochondria. We found shrunk mitochondria with the condensed outer membrane in CCl_4_-treated mice liver (Fig. 2C). In agreement with the hepatoprotective activity, bicyclol and Fer-1 treatment drastically reversed the CCl_4_-induced destruction of mitochondria. Taken together, these data have provided convincing evidence that ferroptosis is responsible for CCl_4_-induced liver injury.

### Bicyclol suppresses CCl_4_-induced cellular ferroptotic cascade

**Fig. 3:**
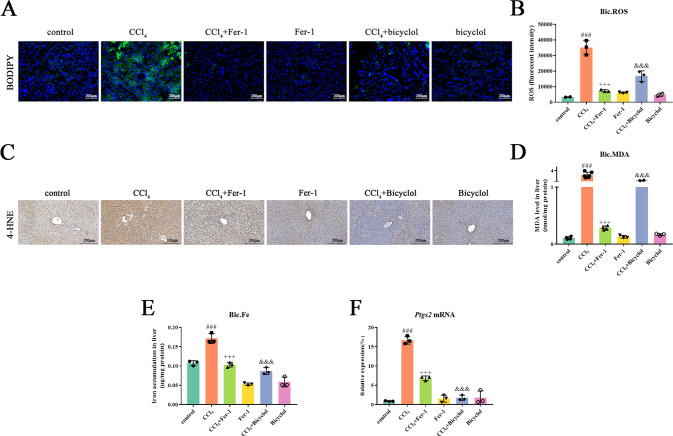
Bicyclol mitigates CCl_4_-induced ferroptosis process in vivo. **A** The accumulation of lipid peroxidation were analyzed by 581/591 C11-BODIPY in liver tissue. **B** Hepatic ROS content was assessed. **C** 4-HNE protein adducts expression was measured in fixed liver tissue sections. **D** The content of MDA in the liver tissues was evaluated. **E** Hepatic iron concentration was assessed. **F** *Ptgs2* mRNA levels were determined by real-time PCR analysis. Data were expressed as mean ± SD. ^###^*p* < 0.001 vs control group, ^+++^*p* < 0.001 vs CCl_4_ group, ^&&&^*p* < 0.001 vs CCl_4_ group.

Iron accumulation, extensive ROS production, and lipid peroxidation are considered to be hallmarks of ferroptosis. Notably, remarkable accumulation of lipid ROS products, which is evaluated by specific fluorescent probe 581/591 C11-BODIPY, was found in response to CCl_4_ administration (Fig. 3A). On the contrary, no lipid ROS accumulation was observed in the control group. Intriguingly, both bicyclol and Fer-1 treatment resulted in a drastic decrease of lipid ROS signals and hepatic ROS accumulation (Fig. 3B). Moreover, reactive toxic aldehydes, including 4-HNE and MDA, which can form covalent adducts with protein, dramatically increased relative to the control group (Fig. 3C, D) [12]. Treatment with bicyclol abrogated the elevated contents of MDA and 4-HNE, and similarly, negative effect was found upon Fer-1 treatment.

Previous studies have demonstrated that iron overload contributes to a wide array of pathological conditions and ferroptosis potentiates the pathogenesis of liver damage [13, 14]. Consistently, we found an increased level of ferrous iron in the liver of CCl_4_-treated mice, whilst both bicyclol and Fer-1 were found to reverse ferrous iron contents (Fig. 3E). Moreover, we examined gene expression of *Ptgs2*, referring to an identified ferroptosis biomarker in vivo [15]. As expected, we showed that bicyclol and Fer-1 diminish *Ptgs2* mRNA levels in the liver of mice exposed to CCl_4_ (Fig. 3F).

### Bicyclol enhances the expression of xCT and GPx4

**Fig. 4:**
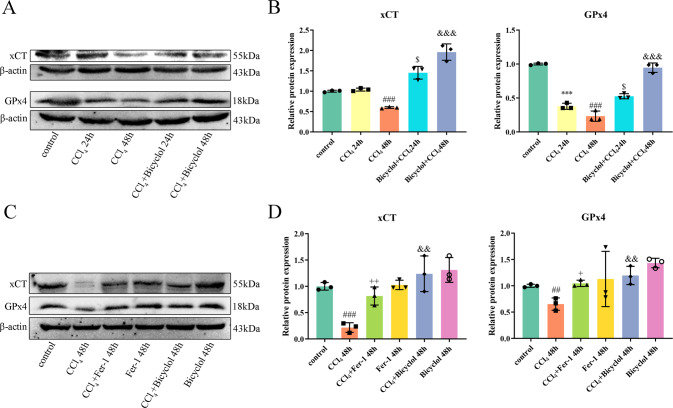
Effects of bicyclol on critical ferroptosis regulators in vivo. **A** Protein levels of xCT and GPx4 in liver tissues at 24 and 48 h after CCl_4_ exposure. **B** The band intensities of xCT and GPx4 relative to β-actin were quantified by image J software. **C** Hepatic protein levels of xCT and GPx4 upon Fer-1 or bicyclol treatment at 48 h after CCl_4_ exposure. **D** The band intensities of xCT and GPx4 relative to β-actin were quantified. Data were expressed as mean ± SD. ^#^*p* < 0.05, ^###^*p* < 0.001 vs control group, ***p < 0.001 vs control group, ^$^*p* < 0.05 vs CCl_4_ 24 h group, ^+^*p* < 0.05, ^+++^*p* < 0.001 vs CCl_4_ 48 h group, ^&&^*p* < 0.01, ^&&&^*p* < 0.001 vs CCl_4_ 48 h group.

As aforementioned, xCT and GPx4 are functioning as two critical regulators in the process of ferroptosis. Therefore, we are intended to detect the perturbations of these proteins. Our data demonstrated that CCl_4_ treatment decreases the protein levels of xCT ~40.1% that of the control at 48 h, whereas bicyclol reversed this effect by increasing 3.3-fold over the CCl_4_ exposure group (Fig. 4A, B). In addition, the expression levels of GPx4 significantly decreased to 37.8% and 23.2% after 24 and 48 h of CCl_4_ exposure, respectively, from that of the control group (Fig. 4A, B). In the CCl_4_ + bicyclol 24 h group, the expression level of GPx4 increased 1.4-fold from that of CCl_4_ exposure group. In the CCl_4_ + bicyclol 48 h group, the expression level of GPx4 increased 4.1-fold from that of CCl_4_ exposure group. Notably, Fer-1 also ameliorated expression levels of xCT and GPx4, which were diminished upon CCl_4_ challenge (Fig. 4C, D). Collectively, these results clearly implicated a pivotal role of cellular ferroptosis in ALI model and the association between hepatoprotective activities of bicyclol and ferroptosis suppression in vivo.

### Bicyclol modulates several key mediators of ferroptosis process

**Fig. 5:**
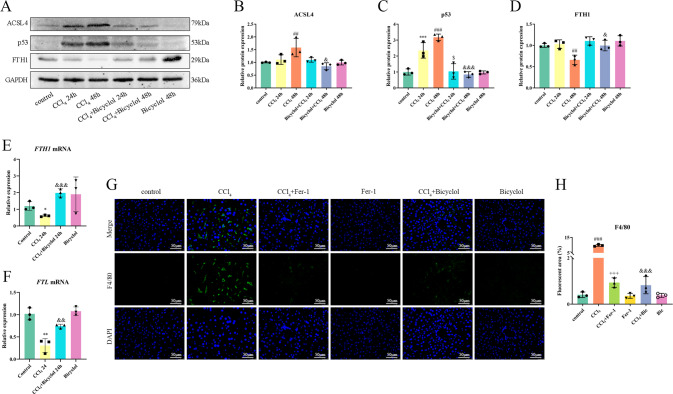
Effects of bicyclol on key mediators of ferroptosis process in vivo. **A** Hepatic protein levels of ACSL4, p53, and FTH1 upon bicyclol treatment at 24 and 48 h after CCl_4_ exposure. **B**–**D** The band intensities of ACSL4, p53, and FTH1 relative to β-actin were quantified by image J software. **E**, **F** *FTH1 and FTL* mRNA levels were determined by real-time PCR analysis. **G** The macrophages were monitored by F4/80 staining in fixed liver tissue sections. **H** Quantification of F4/80 positive cells were shown. Data were expressed as mean ± SD. ^#^*p* < 0.05, ^###^*p* < 0.001 vs control group, ***p < 0.001 vs control group, ^$^*p* < 0.05 vs CCl_4_ 24 h group, ^+++^*p* < 0.001 vs CCl_4_ 48 h group, ^&^*p* < 0.05, ^&&&^*p* < 0.001 vs CCl_4_ 48 h group.

It has been well documented that ferroptosis process can be regulated by multiple molecules from distinct facets. Emerging evidence has indicated that p53-KR interrupts the import of cystine, and ultimately results in GSH depletion and cell death in a ferroptotic manner [16]. Acyl-CoA synthetase long-chain family member 4 (ACSL4) is responsible for the execution of ferroptosis because of its ability to ligate coenzyme A to long-chain polyunsaturated fatty acids (PUFAs). In the case of GPx4 dysfunction, PUFAs located in the cellular membrane may undergo peroxidation, leading to excessive lipid peroxidation and ferroptosis [17]. The expression level of ferritin heavy chain 1 (FTH1) influences sensitivity to ferroptosis, whereas FTH1 silencing exacerbates erastin-induced ferroptosis [18, 19]. Taken together, the expression levels of p53, ACSL4, and FTH1 proteins were assessed to illustrate the medicinal effect of bicyclol against CCl_4_ intoxication in the mice liver. As shown in Fig. 5A–D, the positive mediators of ferroptosis, namely p53 and ACSL4, were significantly upregulated in the CCl_4_ exposure group, whilst the expression level of FTH1 was drastically decreased in the CCl_4_ exposure group at 48 h relative to the control group. Furthermore, treatment of bicyclol noticeably reversed these changes. We then set out to measure inflammatory response and lipid peroxidation as consequences of ferroptosis in our ALI model. Hepatic *FTH1* and *FTL* gene expression were downregulated, and *HMGB1*, *ALOX15,* and *p21* gene expression were upregulated in CCl_4_-injured mice, whereas both bicyclol and Fer-1 treatment reversed these alterations (Fig. 5E, F and Fig. S1). Furthermore, F4/80 positive macrophages were markedly increased in response to CCl_4_ exposure, which was also alleviated by treatment with bicyclol (Fig. 5G, H). Collectively, these data clearly implicated that bicyclol impacts multiple steps of ferroptosis process.

### Bicyclol suppresses erastin-induced cellular ferroptotic cascade

**Fig. 6:**
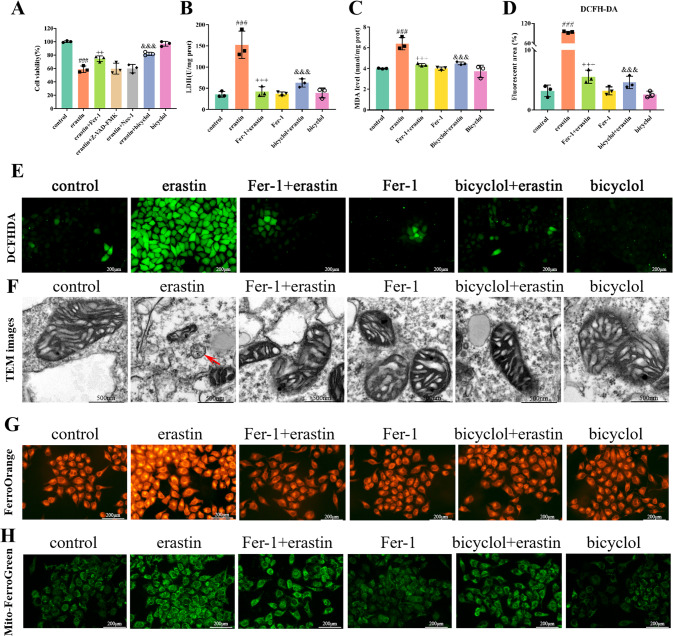
Hepatocellular ferroptosis triggered by erastin is prevented upon bicyclol supplementation. **A** The viability of L-O2 cells challenged with erastin and various specific inhibitors including Z-VAD-FMK, Nec-1, Fer-1, and bicyclol was assessed by CCK-8. **B** The content of LDH in L-O2 cells was evaluated. **C** The content of MDA in L-O2 cells was evaluated. **D** Quantification of cellular ROS content was shown. **E** Cellular ROS content was assessed by DCFH-DA probe. **F** TEM was used to detect the mitochondrial morphology of cultured L-O2 cells upon different treatment. **G** FerroOrange probe was used to detect the intracellular Fe^2+^ of cultured L-O2 cells upon different treatment. **H** Mito-FerroGreen probe was used to detect the mitochondrial Fe^2+^ of cultured L-O2 cells upon different treatment. Data were expressed as mean ± SD. ^###^*p* < 0.001 vs control group, ^++^*p* < 0.01, ^+++^*p* < 0.001 vs erastin group and ^&&&^*p* < 0.001 vs erastin group.

To further verify the responsibility of ferroptosis in liver injury, several inhibitors, including Z-VAD-FMK (10 μM), Nec-1 (10 μM), and Fer-1 (2 μM), were applied to explore the subroutines of RCD in erastin-induced (30 μM) cell death in vitro. Our data indicated that both bicyclol (10 μM) and Fer-1 mitigate erastin-induced cell death to some extent, whilst Z-VAD-FMK and Nec-1 exhibit no such effects (Fig. 6A). Similarly, results showed that both bicyclol and Fer-1 prevent erastin-induced accumulation of LDH and MDA (Fig. 6B, C). Using DCFH-DA probe, we found that bicyclol markedly reduces lipid peroxidation evoked by erastin in L-O2 cells (Fig. 6D, E). Furthermore, we discovered that bicyclol restores dense and shrunken mitochondria induced by erastin in cells according to TEM evaluation (Fig. 6F). Next, we measured the content of cellular Fe^2+^ and mitochondrial Fe^2+^ by using FerroOrange and Mito-FerroGreen probes. As shown in Fig. 6G, H, erastin treatment induced excessive iron content in L-O2 cells as indicated by higher FerroOrange and Mito-FerroGreen signals than that of normal control cells, whilst these changes could be remarkably ameliorated by Fer-1 and bicyclol treatment.

**Fig. 7:**
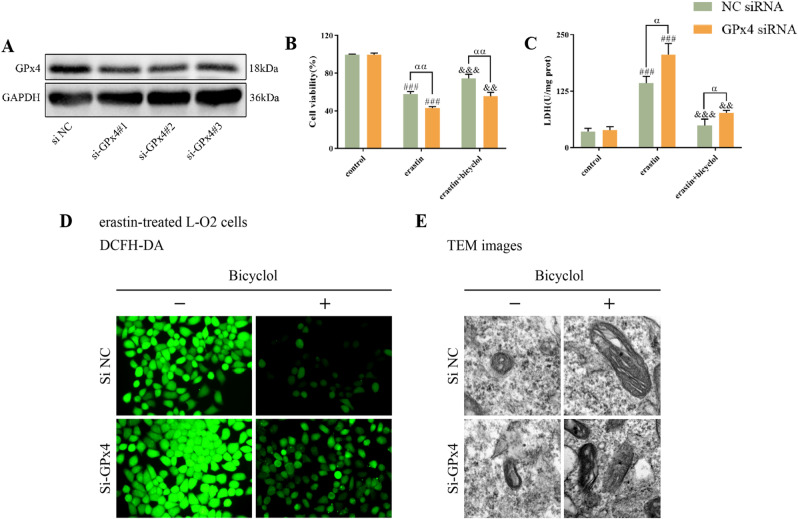
Bicyclol attenuates hepatocellular ferroptosis by regulating GPx4 expression. **A** Protein levels of GPx4 in L-O2 cells transfected with nontargeting control siRNA or GPx4 siRNA. **B** The viability of L-O2 cells transfected with nontargeting control siRNA or GPx4 siRNA upon different treatment was assessed by CCK-8. **C** The content of LDH in L-O2 cells transfected with nontargeting control siRNA or GPx4 siRNA upon different treatment was evaluated by commercial assay kit. **D** Cellular ROS content in L-O2 cells transfected with nontargeting control siRNA (NC siRNA) or GPx4 siRNA was assessed by DCFH-DA probe. **E** TEM was used to detect the mitochondrial morphology of L-O2 cells transfected with NC siRNA or GPx4 siRNA upon different treatment. Data were expressed as mean ± SD. ^###^*p* < 0.001 vs control group, ^&&^*p* < 0.01, ^&&&^*p* < 0.001 vs erastin group, ^α^*p* < 0.05, ^αα^*p* < 0.01 denotes significant difference between siRNA transfected groups.

Furthermore, siRNA-mediated knockdown of GPx4, an intrinsic negative modulator of ferroptosis and lipid peroxidation, was used, and efficient knockdown was registered by western blot (Fig. 7A). GPx4 knockdown significantly enhanced erastin-induced cell death in L-O2 cells, whilst bicyclol exhibited no rescue impact with respect to cell viability and LDH contents (Fig. 7B, C). DCFH-DA and TEM results confirmed the critical role of GPx4 upon bicyclol administration (Fig. 7D, E). Collectively, these in vitro data further revealed the notion that targeting ferroptosis is responsible for the hepatoprotective activities of bicyclol via GPx4 pathway.

### Bicyclol attenuates cellular ferroptosis by regulating Nrf2

**Fig. 8:**
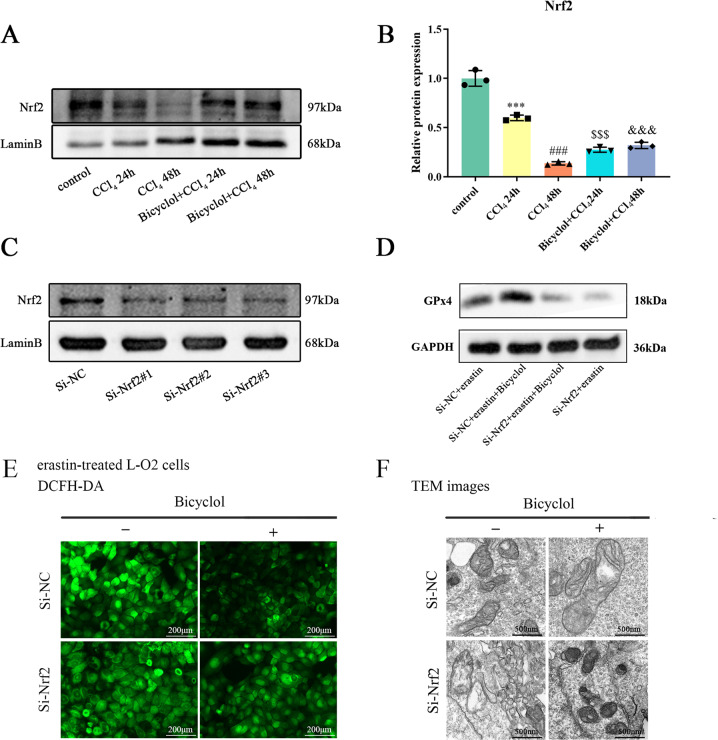
Bicyclol attenuates hepatocellular ferroptosis by regulating Nrf2-GPx4 axis. **A** Nuclear protein levels of Nrf2 in liver tissues at 24 and 48 h after CCl_4_ exposure. **B** The band intensities of Nrf2 relative to LaminB were quantified by image J software. **C** Protein levels of Nrf2 in L-O2 cells transfected with nontargeting control siRNA or Nrf2 siRNA. **D** Protein levels of GPx4 in erastin-treated L-O2 cells transfected with nontargeting control siRNA or GPx4 siRNA. **E** Cellular ROS content in L-O2 cells transfected with nontargeting control siRNA (NC siRNA) or Nrf2 siRNA was assessed by DCFH-DA probe. **F** TEM was used to detect the mitochondrial morphology of L-O2 cells transfected with NC siRNA or Nrf2 siRNA upon different treatment. Data were expressed as mean ± SD. ***p < 0.001 vs control group, ^###^*p* < 0.001 vs control group, ^$$$^*p* < 0.001 vs CCl_4_ 24 h group, ^&&&^*p* < 0.001 vs CCl_4_ 48 h group.

It has been suggested that Nrf2 can protect various organs in the context of distinct pathologies by regulating ferroptosis process. Cumulative evidence has suggested that several natural derivatives represent hepatoprotective potentials by activating Nrf2 signaling pathway [20, 21], and our previous work has proved Nrf2 as a downstream target with respect to the medicinal effect of bicyclol. Therefore, we reasonably hypothesized that the hepatoprotective actions of bicyclol are also relevant to Nrf2 pathway. To prove this hypothesis, we firstly determined the impact of bicyclol on Nrf2 protein expression in CCl_4_-injured mice. As shown in Fig. 8A, B, treatment with bicyclol in the presence of CCl_4_ significantly enhanced nuclear Nrf2 protein expression. Simultaneously, knockdown of Nrf2 by specific siRNA in L-O2 cells was performed to further assess the protective property of bicyclol against erastin-induced hepatotoxicity and its dependence upon Nrf2 stimulation in vitro. Western blot analysis showed that Nrf2 falls dramatically in L-O2 cells transfected with Nrf2 siRNA (Fig. 8C). Moreover, Nrf2 knockdown significantly abrogated GPx4 activation in response to bicyclol supplementation in erastin-treated L-O2 cells (Fig. 8D). Similarly, bicyclol triggered protection was abolished in the deficiency of Nrf2 (Fig. 8E, F), as evidenced by DCFH-DA and TEM results.

Taken together, the current study on the basis of in vivo and in vitro experiments strongly indicates that the positive regulation of Nrf2-GPx4 axis by bicyclol is responsible for its hepatoprotection against ferroptosis process.

## Discussion

The major findings of the current study are as follows: (1) Ferroptosis is involved in CCl_4_-induced ALI, which is characterized by iron accumulation, lipid peroxidation, and aberrant expression of critical modulators. Consistently, these detrimental effects can be remarkably abrogated by Fer-1, namely a specific ferroptosis inhibitor; (2) CCl_4_-induced hepatotoxicity is ameliorated by bicyclol via restoring iron homeostasis and suppressing the instigation and execution of ferroptosis in vivo; (3) Further in vitro results demonstrate that pharmacological regulation of Nrf2 and its transcriptional target GPx4 may prevent ferroptosis process elicited by erastin in L-O2 cells. These findings clarify the molecular machinery underlying CCl_4_-induced hepatotoxicity and suggest that bicyclol represents a viable protective agent by modulating ferroptotic cell death via Nrf2-GPx4 axis.

Bicyclol is a synthetic hepatoprotectant on the basis of the active component schizandrin C, which is extracted from *Schisandra chinensis*. Since its approval by the Chinese Food and Drug Administration in 2004, a gamut of clinical studies and basic research has been conducted to deepen its medicinal effect and underpinning mechanisms. Retrieved literature data imply that anti-inflammatory action, antioxidative stress, elimination of free radicals, and prevention against nuclear DNA damage may in part account for its hepatoprotective mechanisms. More recently, the potentially molecular machinery of bicyclol has expanded into novel facets, for instance, modification of RCD and manipulation of gut microbiota [22, 23]. On the other hand, a nationwide database enrolling 25,927 patients with drug-induced liver injury demonstrated that bicyclol treatment significantly improves the ALT normalization rate when compared with a control group receiving only supportive treatment) [24]. No differences were found regarding renal impairment as well as other adverse events. Collectively, the efficacy and safety of bicyclol merit further in-depth investigation from another pathobiological perspective, such as ferroptosis in the current study.

It has been well documented that CCl_4_-induced hepatotoxicity partially depends on excessive ROS accumulation and lipid peroxidation [25]. Lipid peroxides react with cellular iron, giving rise to some reactive aldehydes, including 4-HNE and MDA. These aldehydes are capable of conjugating to GSH from protein adducts and, in turn, consume the GSH contents. In the current study, we found drastically increased C11-BODIPY staining (a biomarker of ROS production), elevated 4-HNE and MDA in the mice liver tissues at 48 h challenged by CCl_4_, which are all significantly improved by bicyclol supplementation. On the other hand, toxic free radicals following CCl_4_ exposure result in many biological processes (e.g., various types of RCD), such as necrosis, apoptosis, autophagy, and ferroptosis [2]. From a therapeutic perspective, robust cytoprotection may not be obtained by targeting a single RCD subroutine but simultaneously intervening with different RCD modalities. As a matter of fact, our previous work already revealed that bicyclol exerts hepatoprotection by promoting autophagic flux and concomitantly inhibiting oxidative stress. Here, we suppose that bicyclol as an antioxidative and RCD modulator can potentially prevent ferroptosis, thereby ameliorating both liver and cellular destruction. Accordingly, we unraveled that both Fer-1 and bicyclol supplementation lead to remarkable protection against hepatocellular ferroptosis in CCl_4_-induced ALI. Examination of ferroptosis-related biomarkers, including Ptgs2, ACSL4, p53, FTH1, and characteristic morphological changes in the mitochondria suggested that ferroptosis and oxidative stress are markedly regulated by bicyclol. Of note, apoptosis remains the default cell death modality in the presence of intact caspases in hepatocytes [26]. However, various cell death types can coexist, and cells can switch from one death subroutine to another. It is the case that delineates which death subroutine is dominant, or whether there is synergy and the simultaneous activation of multiple pathways in hepatocytes merits to be considered. Accordingly, our in vitro results showed no rescue effect pertaining to Z-VAD-FMK (a pan-caspase inhibitor) or Nec-1 (a necroptosis inhibitor) in erastin-mediated cellular destruction, whereas Fer-1 (a free radical scavenger) and bicyclol exhibit obvious rescue impact.

GPx4 has been documented to be the crucial regulator of ferroptosis process. It can reduce phospholipid hydroperoxides and cholesterol hydroperoxides to their counterparts, therefore interrupting the lipid peroxidation chain reaction [27]. GPx4 deficiency due to conditional depletion results in non-apoptotic cell death in response to massive lipid peroxidation [28]. GPx4 is indispensable for maintaining tissue homeostasis and preventing cell death in the context of various organ/tissue damages [29]. Moreover, mounting evidence has demonstrated that genetic depletion of GPx4 can give rise to ferroptosis in an iron-, MEK-, and ROS-dependent manner [30]. In this study, we observed that GPx4 protein levels fall dramatically on account of CCl_4_ exposure, whilst this deleterious effect is reversed by administrating bicyclol (Fig. 4). Erastin, namely a ferroptosis activator, can suppress the uptake of cystine, and in turn result in GSH depletion and GPx4 inactivation. At the same time, our data indicated that bicyclol restores erastin-induced L-O2 viability and cellular destruction, as evidenced by a decrease in LDH, DCFH-DA, and MDA contents. Additionally, GPx4 silencing accelerated ferroptosis process and ROS production, compared to the NC siRNA-treated cells. Taken together, we speculate that bicyclol exhibits hepatoprotective actions via GPx4 stimulation pathway. However, some cancer cells have been proven to survive in response to genetic depletion of GPx4, suggesting that GPx4 suppression alone may be insufficient to kill certain cancer cell types [31]. An important modulator of not only GPx4 expression but also other marked antioxidant defense system components is Nrf2.

Nrf2 is considered to be the mainstay for controlling antioxidant response since a myriad of its downstream target genes are involved in the maintenance of redox balance in cells. Under normal physiological status, Nrf2 presents with unstability and is rapidly ubiquitinated by Kelch-Like ECH-Associated Protein 1 (Keap-1) into the proteasome degradation pathway. Under stressful conditions, Nrf2 translocates to the nucleus to instigate the transcription of antioxidant response element-containing genes. Moreover, stimulation of Nrf2 can negatively modulate ferroptosis through iron, glutathione, lipid metabolism as well as mitochondrial function [32]. Several key targets whose inhibition initiates ferroptosis, such as GPx4 and FTH1, are well documented to be regulated by Nrf2. We (Fig. 8) and others have reported that CCl_4_ exposure crippled the Nrf2 antioxidant defense system and aggravated oxidative damage in the liver [33, 34]. In a previous study, we unveiled that bicyclol exerts hepatoprotection relying on the activation of Nrf2 as well as its downstream genes HO-1, GSTA-1, and NQO-1. Coincidently, Dai et al. showed that knockdown of Nrf2 exacerbated erastin-induced ferroptotic cell death in human HepG2 cells, implicating a pivotal role of Nrf2 in ferroptosis [35]. In the current study, bicyclol supplementation encountered ferroptosis-induced perturbations in L-O2 cells challenged by erastin (Fig. 8). Consistently, knockdown of Nrf2 by siRNA facilitated erastin-induced cell destruction and partially abrogated the protective effect of bicyclol. Taken together, our findings suggested that bicyclol offers protection against ALI through modulating Nrf2-GPx4 axis and subsequent ferroptosis process.

Intriguingly, our preliminary finding implied that bicyclol also regulates the expression of other crucial mediators of ferroptosis. For instance, xCT (also known as SLC7A11) represents the light chain subunit of the cystine/glutamate antiporter system xc-, which accounts for transporting intracellular glutamate in exchange for cystine to generate GSH. At the basal level, xCT is broadly expressed in normal tissues, such as those of the liver and brain [36]. It has been reported that a multitude of mechanisms, including transcription factors and epigenetic modification, can regulate the expression and activity of xCT. The expression of xCT can be dramatically upregulated in response to different stress conditions (i.e., oxidative stress), and facilitate the cells to restore redox homeostasis and survival [37]. Nrf2 is the main transcription factor involved in the stress-induced transcription of xCT. However, our previous findings denoted that bicyclol can significantly suppress Keap-1-dependent degradation of Nrf2, and Nrf2 remains stable to trigger the transcription of xCT in the current study. On the other hand, xCT has also proved to be negatively regulated by transcription factors where p53 serves as the main transcription factor. p53 deficiency can evidently give rise to the upregulation of xCT [16]. One study further implicated that p53 can inhibit the expression of xCT by dampening the function of Nrf2 [38]. In this study, we found that p53 protein level increases due to CCl4 exposure, whilst bicyclol drastically diminishes its expression (Fig. 5). Taken together, we believe that the synergistic effect of upregulated Nrf2 and downregulated p53, two major transcription factors of xCT under stress conditions, may account for the enhanced expression of xCT.

In conclusion, we clarified that ferroptosis is responsible for CCl_4_-induced ALI in mice, which is characterized by iron accumulation, lipid peroxidation, and aberrant expression of critical modulators. Bicyclol mitigates CCl_4_-induced hepatotoxicity via restoring iron homeostasis and suppressing the instigation and execution of ferroptosis. The underpinning mechanism pertaining to the medicinal effect of bicyclol is partially attributed to the regulation of Nrf2-GPx4 axis, which provides a therapeutic avenue for patients with ALI in clinical practice.

## Materials and methods

### Chemicals and reagents

Bicyclol was provided by the Beijing Union Pharmaceutical Company (Beijing, China) with purity ≥ 99%. CCl_4_ was procured from the Fuyu Chemical Industry Co., Ltd. (Tianjin, China). Erastin (HY-15763) was obtained from the MedChemExpress (Shanghai, China). Ferrostatin-1 (Fer-1, SML0583) was purchased from the Sigma (St. Louis, MO, U.S.). Necrostatin-1 (Nec-1, S8037) and Z-VAD-FMK (S7023) were procured from the Selleck Chemicals (Houston, TX, U.S.).

### Animals

All in vivo experiments on animals were accepted by the Institutional Animal Care and Use Committee at TJMUGH (IRB2021-DWFL-142). Animal research was conducted in accordance with international guidelines. Male C57BL/6 mice aged 6–8 weeks (20–22 g each) were procured from the National Institutes for Food and Drug Control (Beijing, China). Mice were fed in laboratory rooms within a regulated environment of a temperature of 23 ± 2 °C and relative humidity at 50 ± 10%, where a 12 h circadian cycle was adopted. Mice were accommodated for 1 week before conducting experiments and allowed ad libitum to food and water.

The mice were treated with intraperitoneal administration (*i.p*.) of oil (control group) or a mixture of CCl_4_ (50%) and oil (50%) at a dosage of 2 ml/kg body weight. In the bicyclol-treated group, mice accepted administration of 200 mg/kg (using 0.5% carboxymethyl cellulose as solvent) by gavage three times a day 1 h before CCl_4_ exposure, while other groups accepted vehicles of the equal volume. Fer-1 was prepared in DMSO (5 mg/kg), and *i.p*. injected into mice once 1 h before CCl_4_ exposure. The dosage of bicyclol was consistent with our previous work. The mice were then sacrificed to collect liver and serum samples after 24 or 48 h (Fig. 1B).

### Cell culture and siRNA knockdown experiment

Normal human hepatocytes L-O2 were procured from the Cell Bank of the Chinese Academy of Sciences (Shanghai, China) and grown by using Dulbecco’s Modified Eagle Medium (DMEM) with 10% fetal bovine serum, 100 IU/ml penicillin, and 100 mg/ml streptomycin with 5% CO_2_ at 37 °C in a humidified incubator. Hepatocytes were transfected with nontargeting control siRNA (NC siRNA, 80 pmol/ml), siRNA directed against Nrf2 (80 pmol/ml), or siRNA directed against GPx4 (80 pmol/ml) for 48 h through Lipofectamine 3000 instructed by the manufacturer.

### Serum biochemical assay

Serum alanine transaminase (ALT) and aspartate aminotransferase (AST) levels were determined by using an Automated Chemical Analyzer (Hitachi 7080, Hitachi High-Technologies Corporation, Japan) with the analytical assay kits (Kehua Bio-engineering Company, Shanghai, China).

### Histopathology assay

For histopathological analysis, liver tissues from treated mice, as indicated, were fixed in 10% neutral buffered formalin. After that, formalin-fixed, paraffin-embedded samples were cut to produce 5 μm thickness sections and stained with hematoxylin and eosin (H&E). The Knodell score was implemented to ascertain the necroinflammatory process severity.

### Terminal dUTP nick end-labeling (TUNEL) assay

TUNEL assay was implemented to identify DNA fragmentation by using the in situ cell detection kit (Roche, Germany) in accordance with the manufacturer’s protocol. The positively stained cells were captured by using fluorescence microscopy (LEICA DM5000B, Germany). Manual counting was completed to calculate the average number of dead cells in each high power field (×200).

### Transmission electron microscopy (TEM)

We used TEM technique to observe morphological changes in the mitochondria in different treatment groups. The specific protocol has been explicitly described in the literature pertaining to our previous work.

### Reactive oxygen species determination

The levels of total ROS and lipid ROS in the liver were measured by using an oxidation-sensitive fluorescence probe 2’,7’-dichlorofluorescein diacetate (DCFH-DA) kit (E004-1-1, Jiancheng Bioengineering Institute, Nanjing, China) and C11-BODIPY (581/591) staining (Servicebio Technology, Wuhan, China), respectively. To visualize the levels of ROS in hepatocytes, L-O2 cells treated as indicated were incubated with DCFH-DA fluorescence probe, and fluorescence imaging was conducted through fluorescence microscopy (LEICA DM5000B, Germany).

### Lipid peroxidation determination

In order to understand the level of lipid peroxidation in the liver, hepatic homogenates were analyzed for malondialdehyde (MDA) by measuring the content of thiobarbituric acid-reactive substances spectrophotometrically at 535 nm with 1,1,3,3-tetraethoxypropane (Sigma–Aldrich) as the standard in line with the manufacturer’s instruction. On the other hand, 4-hydroxynonenal (4-HNE) adducts were stained and registered by using an anti-4-HNE antibody (ab48506, Abcam, Cambridge, MA, U.S.).

### Iron level detection

The ferrous iron (Fe^2+^) concentration in the liver of mice was determined by Iron Assay Kit (BC4355, Solarbio Science & Technology, Beijing, China) in accordance with the manufacturer’s instruction.

To detect cellular Fe^2+^ and mitochondrial Fe^2+^ contents, FerroOrange and Mito-FerroGreen (F374 and M489, Dojindo Laboratories, Japan) were used according to the manufacturer’s protocol, respectively. L-O2 cells were treated with erastin ± Fer-1 or bicyclol as indicated and stained with a final concentration of 1 μmol/L FerroOrange or 5 μmol/L Mito-FerroGreen for 30 min at 37 °C, respectively. Images were acquired by using fluorescence microscopy (LEICA DM5000B, Germany). The cellular fluorescence intensity was calculated and compared through the Image J software.

### Cell viability determination

Cell viability was assessed by using a CCK-8 kit (CK04, Dojindo Laboratories, Japan). Briefly, L-O2 (2 × 10^3^ cells/well) were seeded in a 96-well plate. The cells, as indicated, were cultured at 37 °C with 5% CO_2_ for 24 h, and thereafter incubated in a complete medium with CCK-8 (0.5 mg/ml) for another 3 h. The density of each well was optically measured at 450 nm by a microplate reader (Tecan Infinite 200pro, Switzerland), and the OD values were read accordingly.

### Quantitative real-time PCR analysis

In liver, total RNA was collected through Trizol reagent (Invitrogen, Carlsbad, CA, U.S.). Totally, 2.5 mg of RNA was reversely transcribed to cDNA by using SuperScript III First-Strand Synthesis System (Invitrogen, Carlsbad, CA, U.S.). Quantitative PCR was conducted by using the ABI PRIAM Step-One Real-time PCR System (Applied Biosystems, Carlsbad, CA, U.S.). A final reaction volume of 20 μl was applied. The amplification conditions were as follows: 50 °C (2 min); 95 °C (5 min); followed by 50 cycles of 95 °C (15 s) and 60 °C (30 s). All primers used are illustrated in Table S1.

### Western blot analysis

Liver tissues or harvested cells, as indicated, were lysed with RIPA buffer complemented with protease inhibitors. The protein concentration was evaluated through the BCA protein assay kit. In brief, a total of 30 μg proteins were separated by 10% SDS-polyacrylamide gel electrophoresis and subsequently transferred to nitrocellulose membranes. Primary antibodies used were anti-xCT (ab37185, Abcam, 1:1000), anti-GPx4 (ab125066, Abcam, 1:1000), anti-ACSL4 (DF12141, Affinity, 1:1000), anti-p53 (2524, CST, 1:1000), anti-FTH1 (DF6278, Affinity, 1:1000), anti-Nrf2 (12721, CST, 1:1000), anti-LaminB (13435, CST, 1:1000), anti-β-actin (3700, CST, 1:1000), and anti-GAPDH (abs100005, Absin, 1:1000). Second antibodies were peroxidase-conjugated goat anti-rabbit and anti-mouse IgG (1:5000) (Zhongshan Golden Bridge Biotechnology, Beijing, China). To visualize the proteomic bands, an enhanced western luminescent detection kit (Vigorous Biotechnology, Beijing, China) was employed. The densitometry results were quantitatively analyzed by using the Image J software, with β-actin, GAPDH, or LaminB bands being normalized/internal controls as appropriate.

### Statistical analysis

All data were demonstrated as mean ± standard deviation (SD). We compared the overall significance of data through two-way analysis of variance. Differences across groups were regarded as statistical significance at *p*-value < 0.05 with the Bonferroni correction for multiple comparisons. GraphPad Prism 8.0.1 (Graph Pad Software, Inc. San Diego, CA, U.S.) was adopted for all analyses.

## Supplementary information

Fig S1

Table S1

Uncropped WB for Fig 4

Uncropped WB for Fig 5

Uncropped WB for Fig 7

Uncropped WB for Fig 8

## Data Availability

Data used and/or analyzed during the current study are available from the corresponding authors on reasonable request.

## Materials availability

Materials used and/or analyzed during the current study are available from the corresponding authors on reasonable request.
