# Supplementary material for: Regulating Nrf2-GPx4 axis by bicyclol can prevent ferroptosis in carbon tetrachloride-induced acute liver injury in mice
Source: Cell Death Discov. 2022 Sep 7;8:380. doi: 10.1038/s41420-022-01173-4 (PMC9452542; doi:10.1038/s41420-022-01173-4)
Supplement: Supplementary file 1 — Fig S1 [file 41420_2022_1173_MOESM1_ESM.docx]

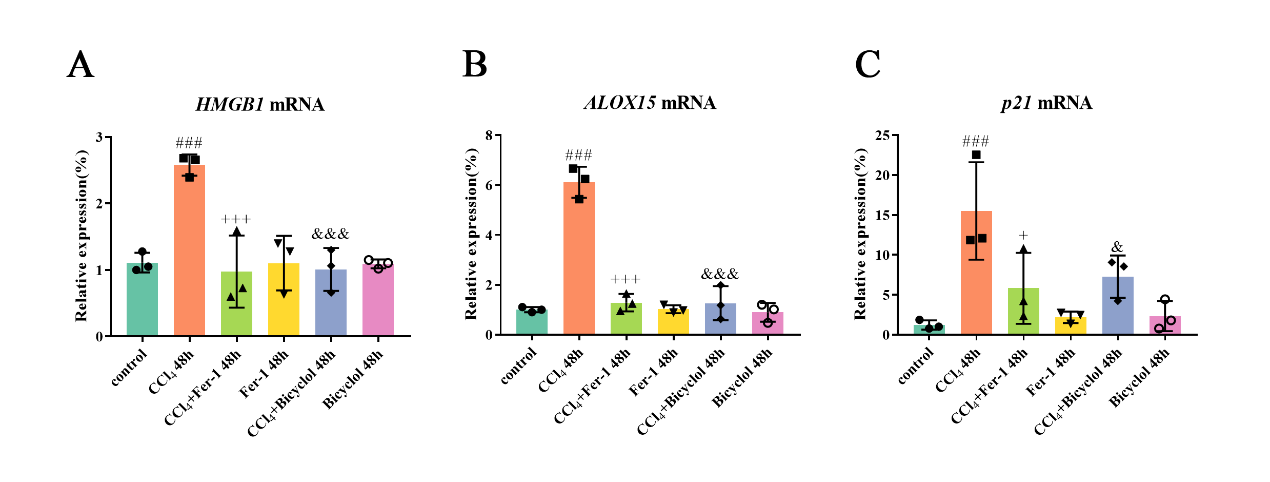


**Fig S1** *HMGB1*, *ALOX15* and *p21* mRNA levels were determined by real-time PCR analysis. Data were expressed as mean ± SD. ###p < 0.001 *vs* control group, +++p < 0.001 *vs* CCl_4_ group, &&&p < 0.001 *vs* CCl_4_ group.
