# Supplementary material for: Regulating Nrf2-GPx4 axis by bicyclol can prevent ferroptosis in carbon tetrachloride-induced acute liver injury in mice
Source: Cell Death Discov. 2022 Sep 7;8:380. doi: 10.1038/s41420-022-01173-4 (PMC9452542; doi:10.1038/s41420-022-01173-4)
Supplement: Supplementary file 2 — Table S1 [file 41420_2022_1173_MOESM2_ESM.docx]

**Table S1** List of primers for real-time PCR

| Target | Primer | Sequence |
| --- | --- | --- |
| Ptgs2 | FP | 5’- TGCACTATGGTTACAAAAGCTGG -3’ |
|  | RP | 5’- TCAGGAAGCTCCTTATTTCCCTT -3’ |
| HMGB1 | FP | 5’- GCTGACAAGGCTCGTTATGAA -3’ |
|  | RP | 5’- CCTTTGATTTTGGGGCGGTA -3’ |
| p21 | FP | 5’- CCTGGTGATGTCCGACCTG -3’ |
|  | RP | 5’- CCATGAGCGCATCGCAATC -3’ |
| ALOX15 | FP | 5’- GGCTCCAACAACGAGGTCTAC -3’ |
|  | RP | 5’- CCCAAGGTATTCTGACACATCC -3’ |
| GAPDH | FP | 5’- GGAGAAACCTGCCAAGTATG -3’ |
|  | RP | 5’- TGGGAGTTGCTGTTGAAGTC -3’ |
| FTH1 | FP | 5’-TGCCTCCTACGTCTATCTGTC -3’ |
|  | RP | 5’-GTCATCACGGTCTGGTTTCTTT-3’ |
| FTL | FP | 5’-AGGGCGTAGGCCACTTCTT-3’ |
|  | RP | 5’-CTGGGTTTTACCCCATTCATCTT-3’ |

FP, Forward Primer; RP, Reverse Primer.
