# Supplementary figures and images for: Regulating Nrf2-GPx4 axis by bicyclol can prevent ferroptosis in carbon tetrachloride-induced acute liver injury in mice
Source: Cell Death Discov. 2022 Sep 7;8:380. doi: 10.1038/s41420-022-01173-4 (PMC9452542; doi:10.1038/s41420-022-01173-4)

Fig. 4A β-actin


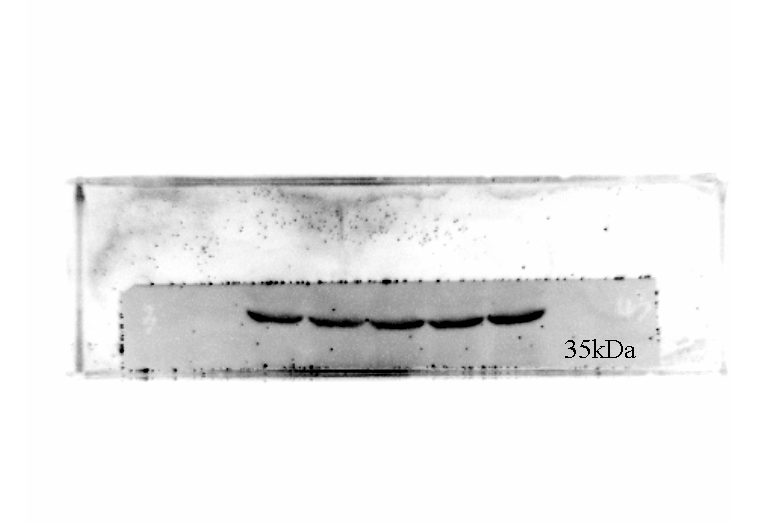


Fig. 4A GPx4


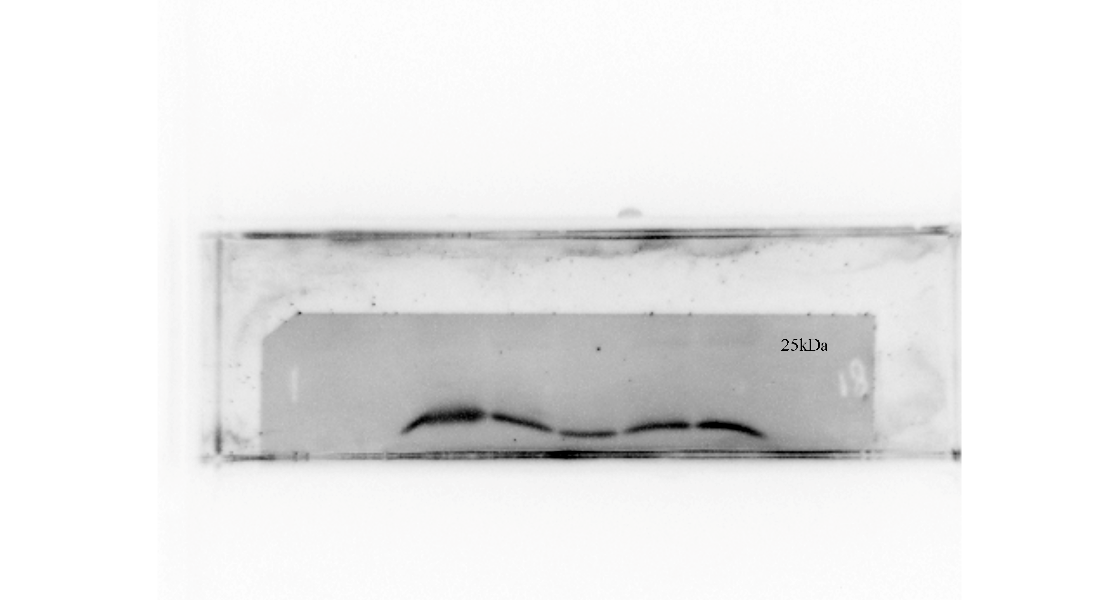


Fig. 4A β-actin


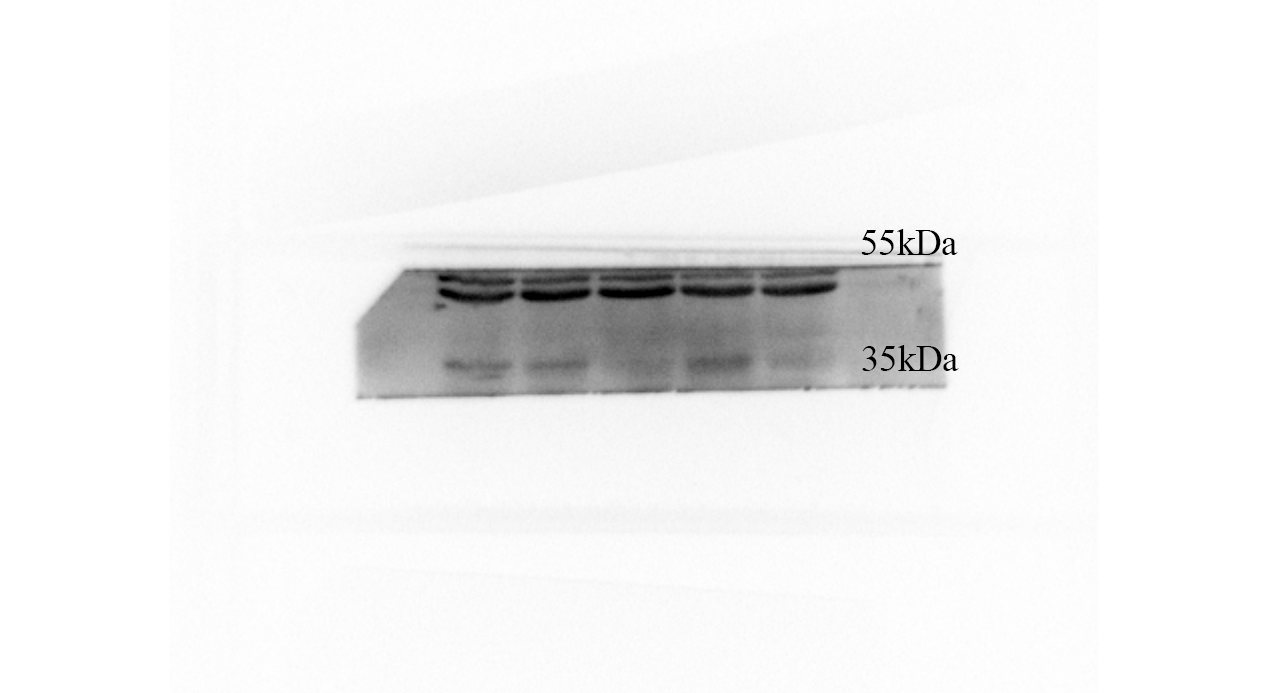


Fig. 4A xCT


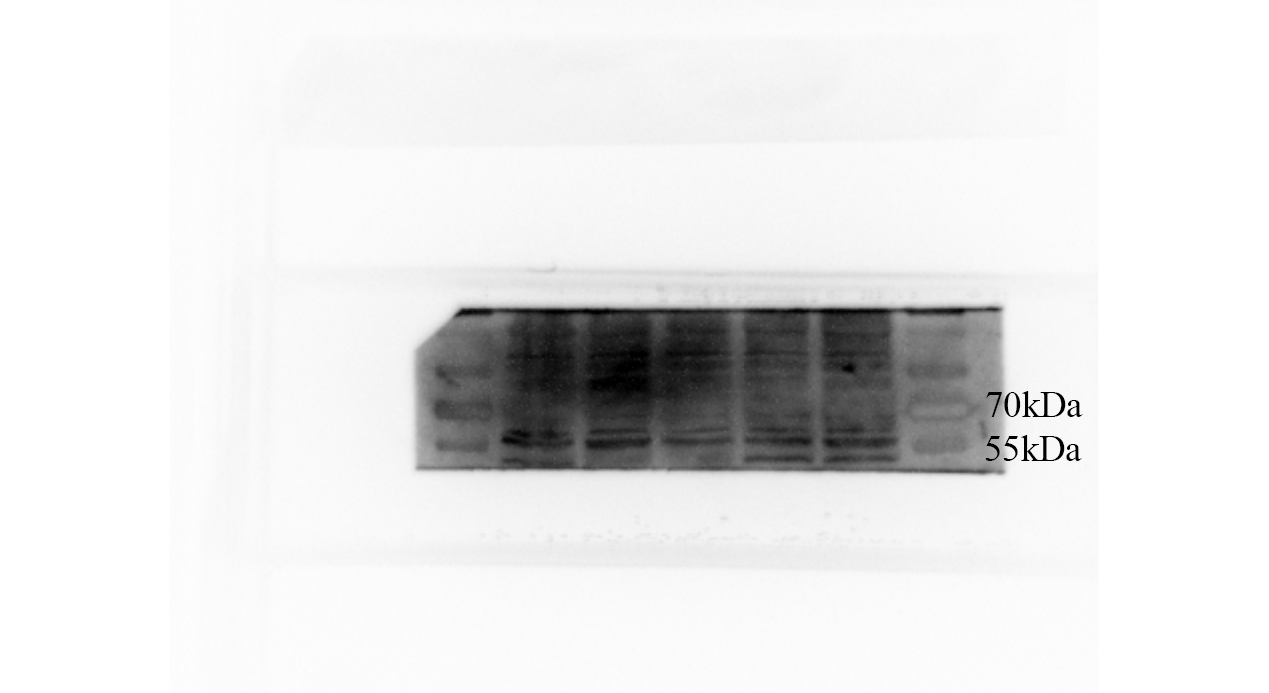


Fig. 4C β-actin


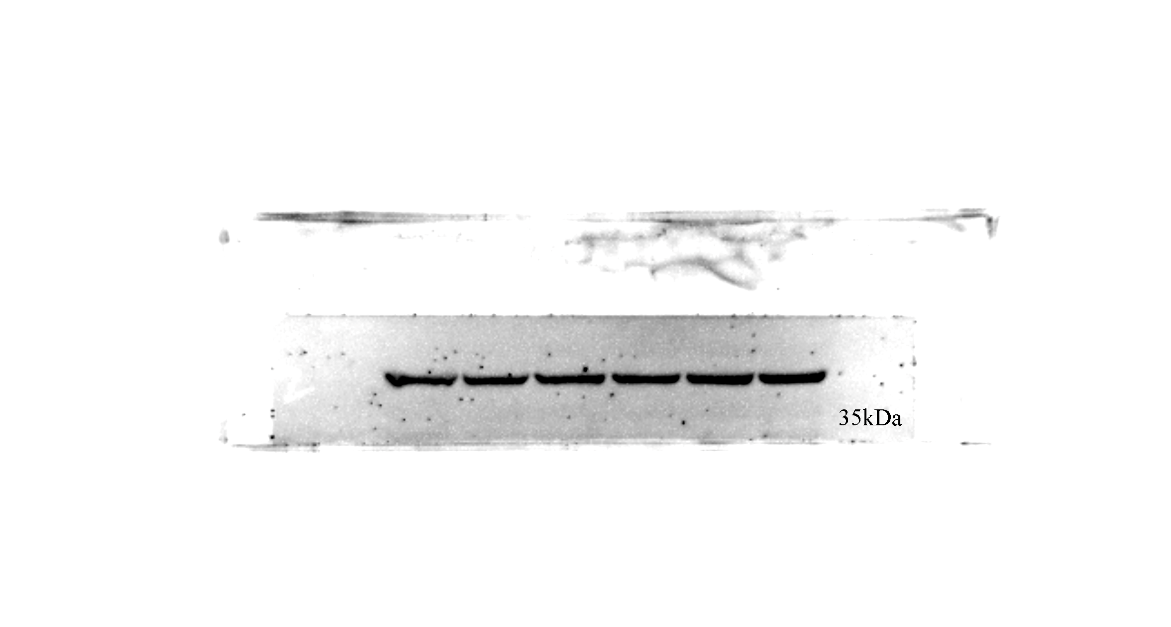


Fig.4C GPx4


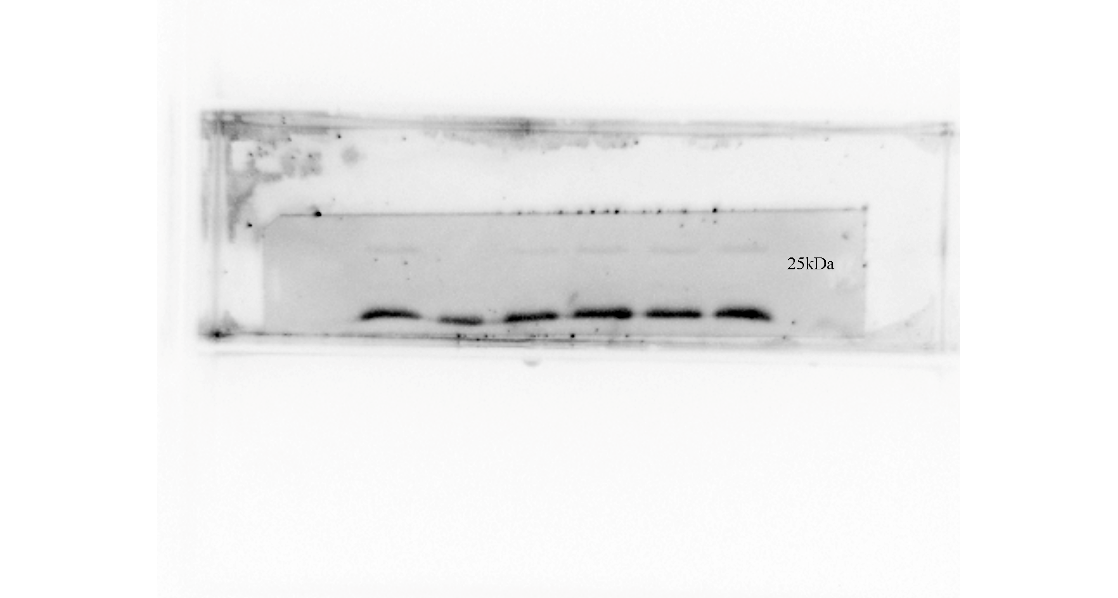


Fig.4C xCT


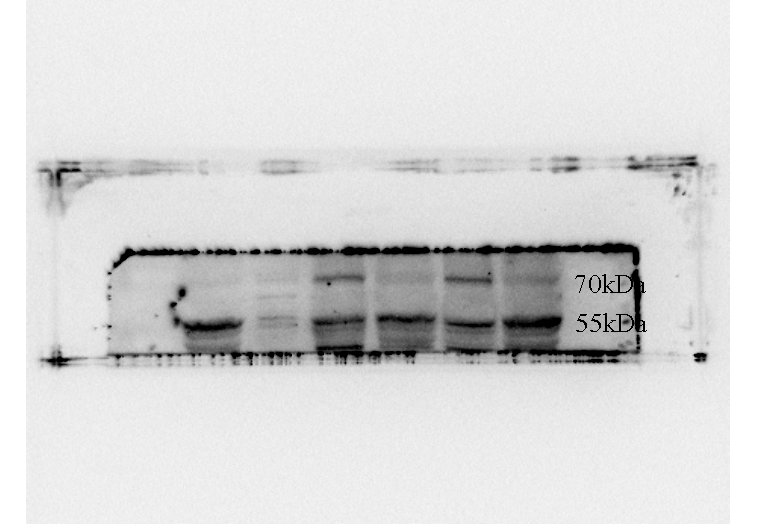

Supplement: Supplementary file 3 — Uncropped WB for Fig 4 [file 41420_2022_1173_MOESM3_ESM.docx]

Fig. 5A ACSL4


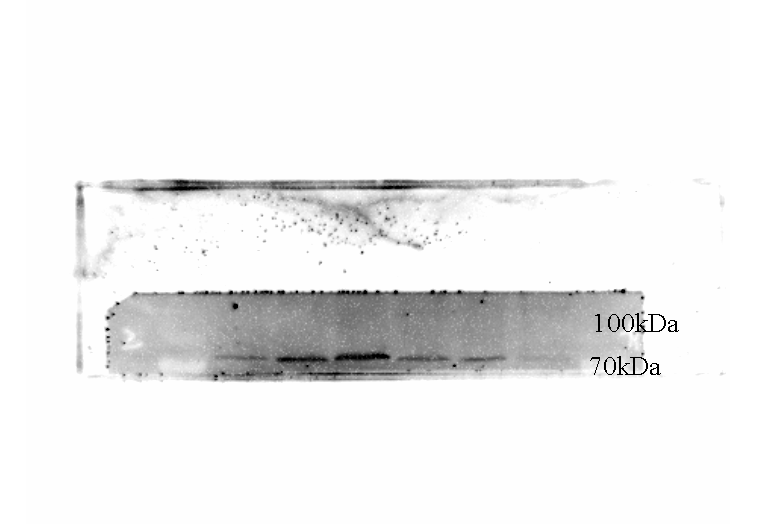


Fig. 5A p53


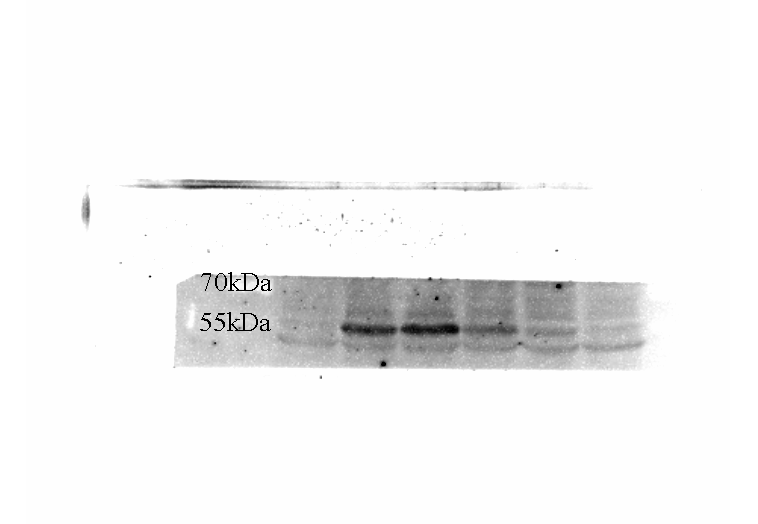


Fig. 5A FTH1


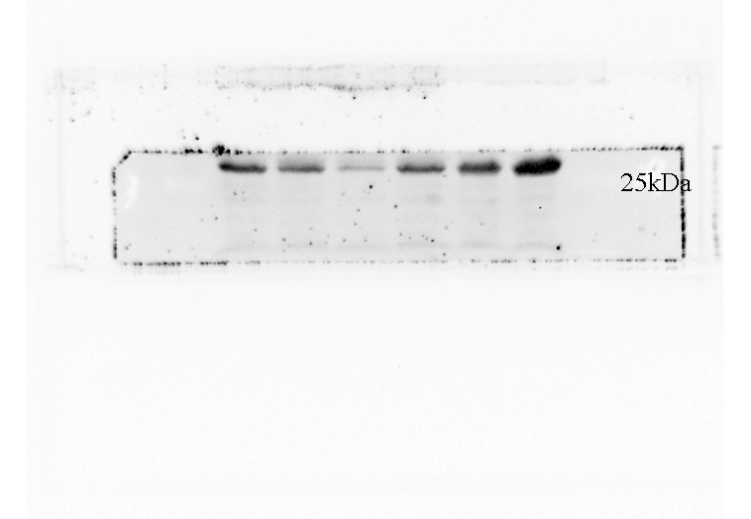


Fig. 5A GAPDH


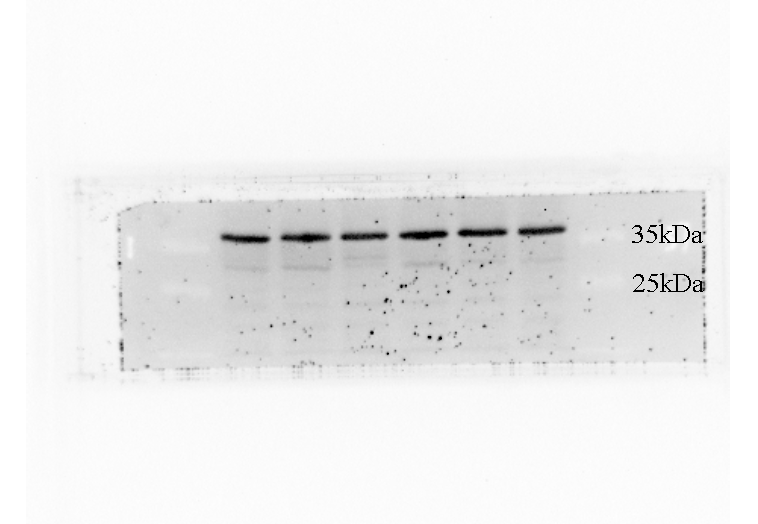

Supplement: Supplementary file 4 — Uncropped WB for Fig 5 [file 41420_2022_1173_MOESM4_ESM.docx]

Fig. 7A

GPx4-GAPDH


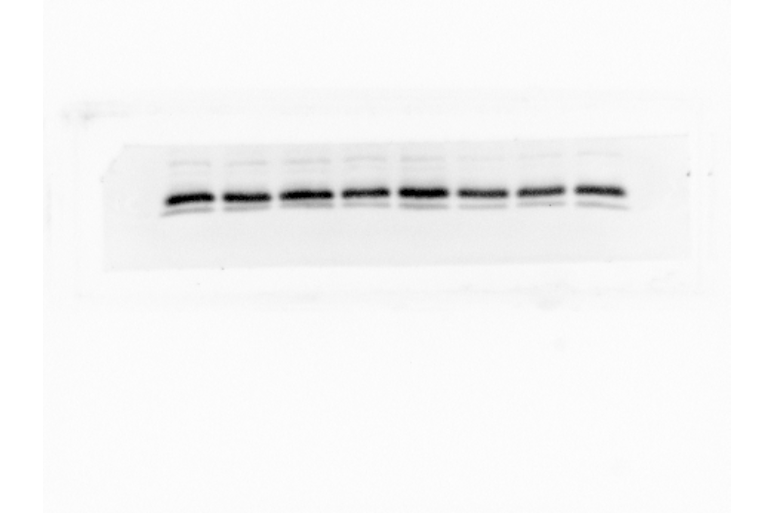


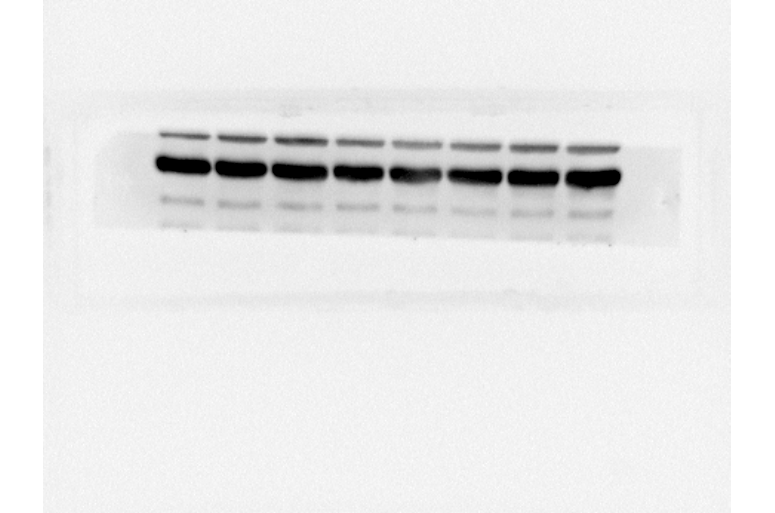

Supplement: Supplementary file 5 — Uncropped WB for Fig 7 [file 41420_2022_1173_MOESM5_ESM.docx]

Fig. 8A

Nrf2-LaminB


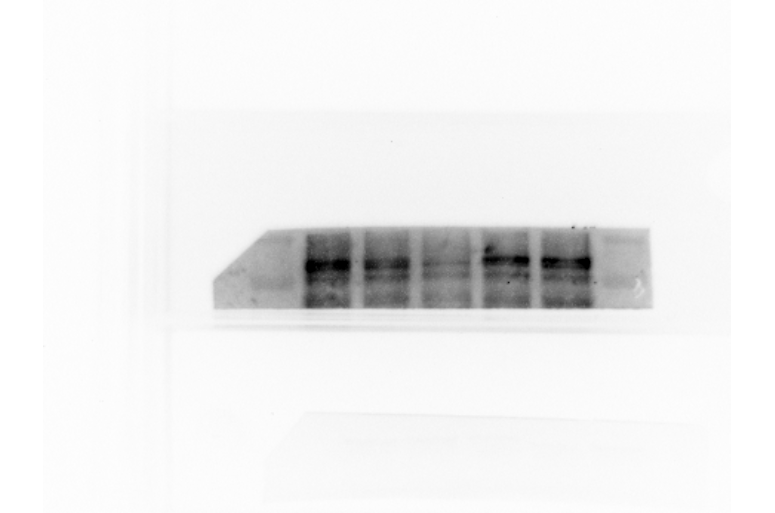


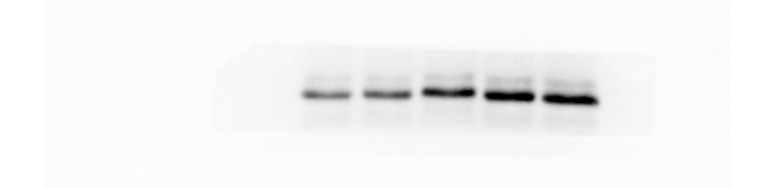


Fig. 8C

Nrf2-LaminB


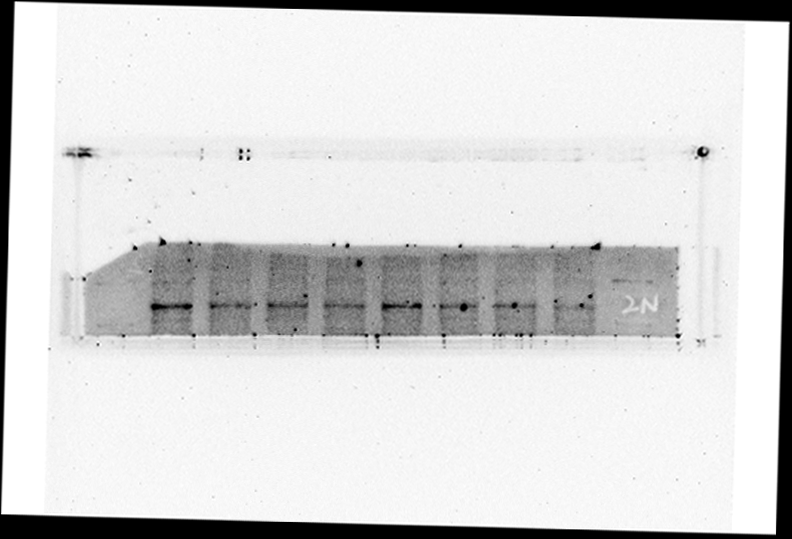


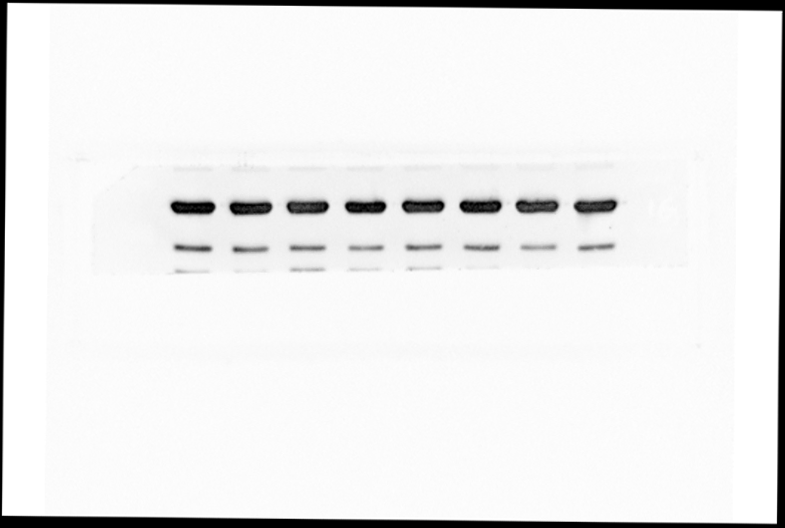


Fig. 8D


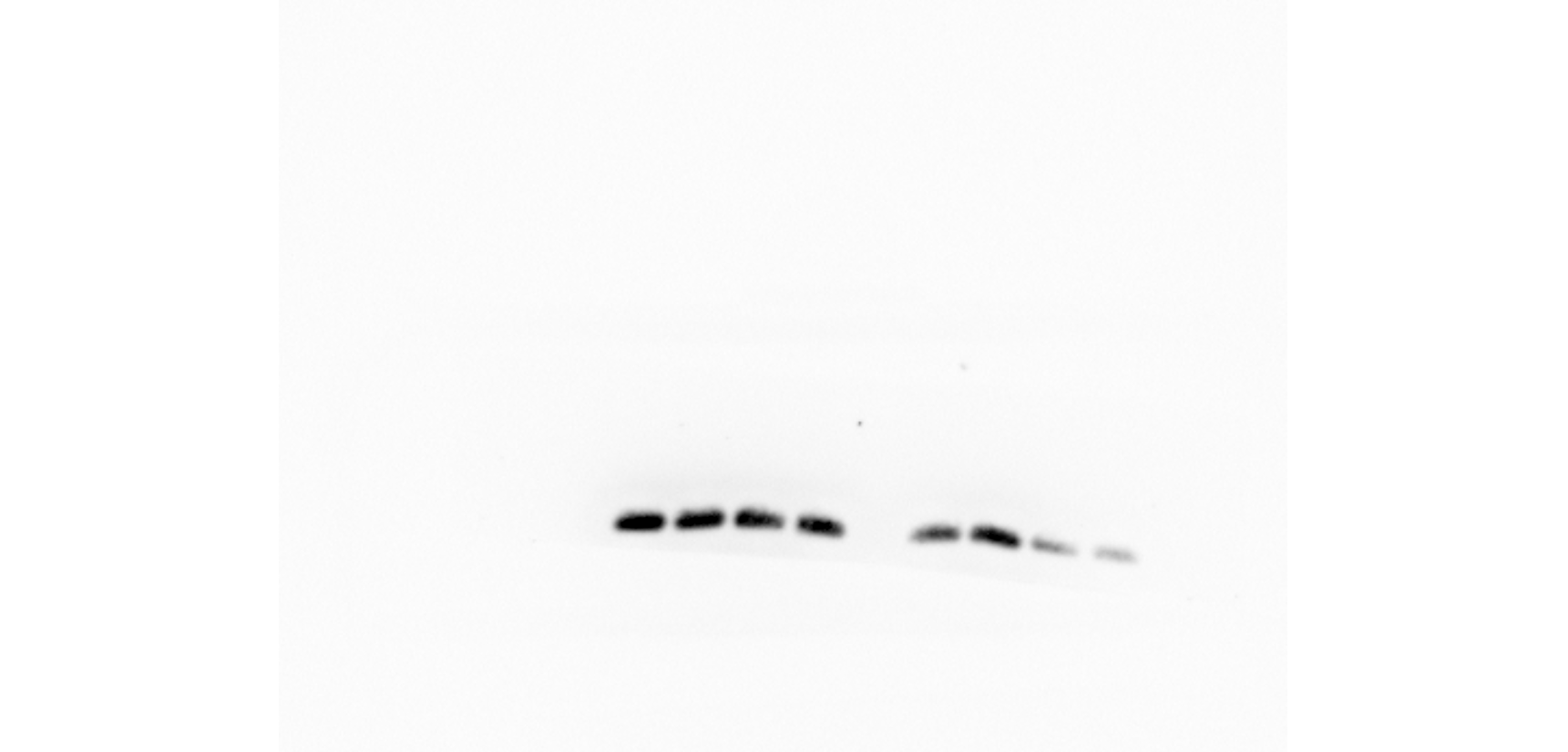
GPx4-GAPDH


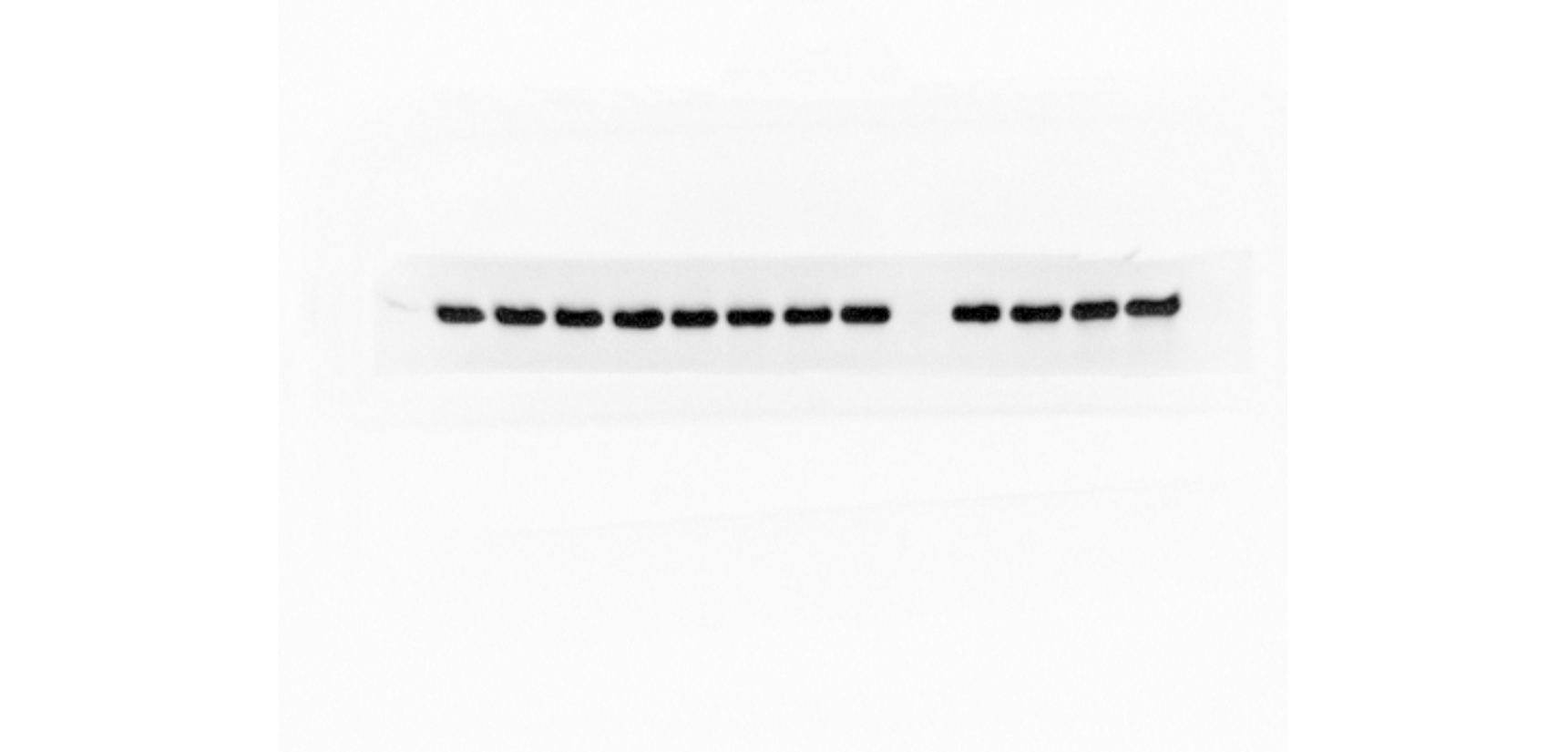

Supplement: Supplementary file 6 — Uncropped WB for Fig 8 [file 41420_2022_1173_MOESM6_ESM.docx]
